# Parental burnout, depression and emotional development of the preschoolers

**DOI:** 10.3389/fpsyg.2023.1207569

**Published:** 2023-06-20

**Authors:** Vera Yakupova, Anna Suarez

**Affiliations:** Faculty of Psychology, Lomonosov Moscow State University, Moscow, Russia

**Keywords:** parental burnout, depression, maternal depression, parenthood, emotion comprehension, emotional development

## Abstract

**Introduction:**

Parental burnout is becoming more and more prevalent in the world, mainly incultures with high demands towards parents. Parental burnout is distinctive from depression and might have its unique influence on child development, which isunder current international research. This work contributes to the understanding of parental burnout, maternal depression and child emotional development(specifically emotion comprehension) interrelations. Additionally, we explored whether there are differences in the effects of parental burnout and depressionon boys and girls.

**Methods:**

To analyse the emotional development of the preschoolers, the Russian version of the Test of Emotional Comprehension (TEC) was used. We used the Russian version of the Parental Burnout Inventory (PBI) to analyse the level of PB and the Russian version of Beck depression Inventory (BDI) to assess participants’ depression level.

**Results:**

Parental burnout positively correlates with child emotional comprehension skills, specifically understanding of external causes (*B* = 0.20, CI: 0.03; 0.37) and mental causes of emotions (*B* = 0.22, CI: 0.05; 0.40). This effect is gender dependent and is significantlyhigher for girls (*B* = 0.54, CI: 0.09; 0.98). The effect of maternal depression on emotion comprehension skills is also gender dependent: total scores on emotioncomprehension tasks are significantly higher for daughters of mothers with depression (*B* = 0.59, CI: 0.001; 1.18).

**Discussion:**

Maternal depression and parental burnout might provoke development of extra sensitivity and self-regulation strategies in girls.

## 1. Introduction

Parenting can be one of the most challenging jobs, with demands for high emotional involvement, multitasking, and many physical and financial resources. This job does not include sick leave, opportunity to quit, or even weekends. At the same time, societal demands to the standards of parenting have increased significantly in the past decades (Roskam et al., 2021), which may lead to parental burnout (PB). PB is commonly defined as a state of intense emotional exhaustion related to one’s parental role, which includes emotional detachment from one’s children and doubts in one’s capacity to be a good parent (Roskam et al., 2017).

Risk factors for PB include child chronic disease, lack of social and family support, high social demands toward parents, conflicts with the spouse and co-parenting problems, emotional dysregulation of a parent and child-oriented perfectionism (Roskam et al., 2021; Favez et al., 2022; Lin et al., 2022a,b; Wauters et al., 2022). The cross-cultural study across 42 countries showed that the main risk factors for PB were lack of support from extended family and high societal demands towards parents, namely, the expectation that parents could control physical, emotional, intellectual and social development of the child as well as minimise any risks for it (Roskam et al., 2021).The authors further showed that PB is common in the majority of countries around the world, with particularly high prevalence in the countries with individualistic cultural values and the trend for intensive parenting (Roskam et al., 2021). However, PB is mainly discussed in relation to parents of children with chronic diseases and/or special needs (Lindström et al., 2011; Gerber et al., 2021; Wauters et al., 2022), while cohort and population-based studies on the topic remain scarce.

This may be partially due to the fact that some symptoms of PB overlap with depression, e.g., sleep problems, escape ideation, and emotional exhaustion (Lebert-Charron et al., 2018; Mikolajczak et al., 2019). However, there is convincing evidence that PB is distinctive from depressive symptomatology and it might have unique consequences for the parents and their children (Sánchez-Rodríguez et al., 2019; Mikolajczak et al., 2020). The surge of studies of the association between PB and child mental health during the COVID-19 pandemic, when there was an increased pressure on parents, revealed that PB was positively associated with children’s stress behaviours, and negatively associated with children’s positive behaviours (Kerr et al., 2021; Koerber et al., 2023).

Nevertheless, to date this topic remains understudied and the existing literature has mainly focused on maternal depression and its consequences for child development. The meta-analysis of 191 studies showed that maternal perinatal depression and anxiety were associated with poorer child’s social–emotional, language and cognitive development in infancy, childhood and adolescence (Rogers et al., 2020). In the Norwegian longitudinal study child social–emotional problems at age 2 were strongly associated both with maternal depression during pregnancy and 8 weeks postpartum (Junge et al., 2017). The study of Abdollahi et al. (2017) indicated that developmental disabilities in communication and gross motor skills in 4 years-old children were associated with concurrent and chronic maternal depressive symptoms, rather than with the perinatal maternal depression. The study of 1,992 mother–child dyads also showed the significance of the current maternal depressive symptoms for the child’s emotional development and communication skills (Hentges et al., 2020).

Findings on gender differences and maternal depression consequences are contradictory. The US longitudinal study showed that maternal depression was associated with poorer social-emotional child development, particularly among girls (Urizar and Muñoz, 2022). A recent meta-analysis indicates that maternal depression affects cognitive development in boys, but not girls (Ahun et al., 2021). The Norwegian longitudinal study showed that while maternal depression affected both infant boys and girls’ cognitive development, girls’ scores tended to increase during the time of observation (Azak, 2012). The study conducted on the adolescent sample revealed that the relationship between maternal depression and child internalising symptoms was significantly larger for daughters compared to sons (Livings, 2021). Such data inconsistency on gender differences in child outcomes could be largely explained by cultural differences and measurement diversity (Ahun et al., 2021) and demonstrates a clear knowledge gap in this area.

Cumulatively, the abovementioned findings indicate that the areas of child development most vulnerable for the effects of parental depression are those related to emotional development. While the consequences of PB for the child’s emotional development remain unknown, there is data showing that PB is associated with parental neglect and violence more often than depression (Mikolajczak et al., 2020; Griffith, 2022), which, in turn, may be damaging for the child emotional development. Furthermore, there is evidence of association between PB, perceived parental hostility and adolescent later externalising problems (Chen et al., 2022). Although a number of studies have also found the association between PB and adolescent behavioural problems (Yuan et al., 2022), student’s problematic behaviour, and academic outcomes (Hong et al., 2022), the direction of these associations remains unclear. To date there is no evidence on gender differences in the PB effects on child emotional development.

Some data suggests that parents of younger age children are at higher risk for PB (Favez et al., 2022). Early childhood is a critical time for social and emotional development: approximately by age 4 children are able to recognize and name emotions and begin to understand how external causes can affect emotions of other people (Pons et al., 2004). High comprehension of emotions performance predicts academic success in preschool children (Da Glória Franco et al., 2017; Garrett-Peters et al., 2017; Józsa and Barrett, 2018; Cavadini et al., 2021). Cultural-historical theory considers understanding of emotions as an important component of any successful child activity (Zaporozhets et al., 1985, p. 26–28). Emotion comprehension plays a significant role in prosocial skills development (Liao et al., 2014; Da Glória Franco et al., 2017). Good understanding of emotions skills are also associated with lower risk of mental health problems (Herba and Phillips, 2004; Martinsen et al., 2019).

Emotional development during the first 3 years of life is strongly associated with social–emotional development at 5 years old (Wang et al., 2022). Maternal depression and anxiety during toddlerhood are also strongly associated with child outcomes at age 5 (Hentges et al., 2020). As there are more tools available to accurately measure emotional development in preschoolers, than in children of 0–3 years old, hence this age group was selected as a cohort for the present study.

Therefore, the aim of this work is to examine the association between PB, depression and child emotional development. We are going to focus on children of preschool age, as it is a sensitive period for emotional development (Pons et al., 2004). Moreover, we are going to compare the effects of maternal concurrent depression and PB on 4 dimensions of the emotion comprehension ability in preschoolers: understanding of emotions caused by external causes, understanding of mental emotions, understanding of the ability to regulate emotions, and the general ability to understand emotions. Finally, we are going to explore whether there are differences in the effects of parental PB and depression on boys and girls.

## 2. Materials and methods

### 2.1. Procedure and participants

The study included two stages: (1) a survey for parents that assessed parental depression and PB and (2) observational diagnostics of the preschoolers’ emotional development. The data was collected during the period of April–June 2022 on the basis of kindergartens in 4 regions of Russia (Yakutia, Tatarstan, Perm, Moscow). The findings are based on the responses of a sample of 251 dyads: parents (*M* = 33.74, SD = 5.08) and children of preschool age (*M* = 4.92, SD = 0.44). The participants received an invitation to take part in the study through the kindergarten’s administration. Families were included in the study if the participating parent was living together with the child and was her legal representative, and the parent was able to speak and read in Russian.

### 2.2. Ethical considerations

Both stages of the study were approved by the Ethical Committee of the Russian Psychological Society, Lomonosov Moscow State University. All participants were asked to sign an informed consent using the online form before starting the completion of the parental survey. The participants signed the agreement for their children to participate in the study. The study was conducted in accordance with the Declaration of Helsinki. All women participated voluntarily in the study. The assessments were carried out with the Testograph online platform.

### 2.3. Measures

#### 2.3.1. The demographic questionnaire

The survey included questions regarding the participants’ age at the time of testing, education (primary/secondary/tertiary) and marital status (married/cohabiting with partner/single). We also collected the data about the child’s age, sex and chronic diseases (reported by the parents).

#### 2.3.2. Beck depression inventory

We used the Russian version of the Beck depression inventory (BDI) to assess the levels of parental depression (Yakupova, 2018). BDI is a 21-item, self-report questionnaire measuring symptoms of depression (Beck et al., 1961). Items are scored on a scale from 0 to 3, e.g., 0: “I do not feel guilty,” 1: “I often feel guilty” 2: “I feel guilty most of the time,” 3: “I feel guilty all the time” The depression score is obtained by summing the 21 item scores. For the Russian version Cronbach’s coefficient is *α* = 0.866 (Yakupova, 2018).

#### 2.3.3. Parental burnout

We used the Russian version of the parental burnout inventory (PBI) (Starchenkova, 2019) to analyse the level of PB in mothers. This is a 23-item questionnaire assessing the four core symptoms of parental burnout: emotional exhaustion (9 items; e.g., I feel completely run down by my role as a parent), contrast with previous parental self (6 items; e.g., I tell myself I’m no longer the parent I used to be), loss of pleasure in one’s parental role (5 items; e.g., I do not enjoy being with my children), and emotional distancing from one’s children (3 items; e.g., I am no longer able to show my children that I love them) (Roskam et al., 2018). The participants were asked to assess each statement using a seven-point frequency scale from 0 to 6 (never, a few times a year, once a month or less, a few times a month, once a week, a few times a week, every day). The parental burnout score is computed by summing the item scores: higher scores reflect higher parental burnout levels. The internal consistency of the Russian version of the scale was Cronbach’s alpha *α* = 0.97 (Roskam et al., 2021).

#### 2.3.4. Child emotional development

To analyse the emotional development of the preschoolers, the Russian version of the test of emotional comprehension (TEC) was used (Pons and Harris, 2000; Veraksa et al., 2021). This test is designed to assess the emotion comprehension skills in children of 3–11 years old. The child is presented with 23 simple plot pictures about the emotional experience of children of the same sex with him. Each plot includes four possible emotional responses. The child needs to evaluate what the protagonist of the story felt in the situation presented. For example: “This boy (girl) is getting a birthday present. How is this boy (girl) feeling? Is he (she) happy, sad, alright or scared?” The test captures information about the different components of emotion comprehension: understanding of emotions caused by external causes, understanding of mental emotions, understanding of the ability to regulate emotions, as well as the general ability to understand emotions. Each answer is scored “0,” if it is wrong, “1” if it is correct. The internal consistency of the Russian version of the test is *α* = 0.97.

#### 2.3.5. Covariates

Two statistical models were used to test our hypotheses. Model 1 was adjusted for child age at testing and sex. Model 2 was further adjusted for maternal age at testing, level of education, family status, socioeconomic status, and parity as covariates. When testing the hypothesis of differential effects of parental burnout and depression on boys and girls, child sex was removed from the list of covariates, as in this analysis the sample was already stratified by sex.

### 2.4. Statistical analysis

BDI and PBI scores were log-transformed to attain normality.

Spearman’s correlation coefficient was used to estimate the relationship between BDI and PBI scores.

Pearson chi-square test and independent *t*-test were performed to compare the sample characteristics between boys and girls. *t*-tests exploring the differences in emotional development were weighted by age.

Multiple linear regression analysis examined the association between parental depression and PB and child emotional development.

All analyses were performed using SPSS 28 software (IBM).

## 3. Results

Demographic characteristics for the parents and their children are presented in Table 1. It shows that the majority of the dyads come from middle-income families (69.7%), where mothers were married (81.6%) and had higher education (61.8%). Furthermore, shows that when comparing boys (*n* = 106) and girls (*n* = 144), boys were slightly older, scored statistically significantly higher on TEC External scale and lower on TEC Mental scale. There were no other significant differences between the subsamples divided according to child sex (*p*-values for all >0.029) (Table 2).

**Table 1:** Characteristics of the sample.

Characteristics		*N* (total)	Mean/*N*	SD/%	Range
Parental characteristics					
Age (years)		227	33.74	5.08	22–48
Sex	Female	251	251	100%	
Education	General secondary/vocational education	228	87	38.2%	
	Higher education		141	61.8%	
Family status	Married	228	186	81.6%	
	Have a partner		16	7.0%	
	Single		26	11.4%	
SES	Low	228	42	18.4	
	Middle		159	69.7	
	High		27	11.8	
BDI total score (raw)		251	5.16	7.27	0–54
PBI total score (raw)		228	10.16	18.08	0–120
Child characteristics					
Age at testing (years)		251	4.92	0.44	3–7
Sex	Female	250	144	57.6%	
	Male		106	42.4%	
Parity	1	228	67	29.4%	
	2		79	34.6%	
	3+		82	36.0%	
TEC external scale		240	2.31	0.73	0–3
TEC mental scale		241	0.84	0.76	0–3
TEC reflective scale		241	1.03	0.80	0–3
TEC total score		241	4.18	1.52	0–8

SES, socioeconomic status; BDI, Beck depression inventory; PBI, parental burnout inventory; TEC, test of emotional comprehension.

**Table 2:** Characteristics of the sample according to child’s sex.

Characteristics		Boys (*n* = 106)			Girls (*n* = 144)			*p*
		Mean/*N*	SD/%	Range	Mean/*N*	SD/%	Range	
Parental characteristics								
Age (years)		33.29	4.96	22–48	34.02	5.15	22–46	0.29
Sex		106	100%		144	100%		
Education	General secondary/vocational education	38	39.6%		48	36.6%		0.68
	Higher education	58	60.4%		83	63.4%		
Family status	Married	80	83.3%		105	80.2%		0.83
	Have a Partner	6	6.3%		10	7.6%		
	Single	10	10.4%		16	12.2%		
SES	Low	16	16.7%		26	19.8%		0.71
	Middle	67	69.8%		91	69.5%		
	High	13	13.5%		14	10.7%		
BDI total score (raw)		5.08	7.20	0–54	5.26	7.36		0.85
PBI total score (raw)		8.79	15.92	0–120	11.24	19.55		0.31
Child characteristics								
Age at testing (years)		4.99	0.40	4–6	4.86	0.45	3–7	0.020
Parity	1	31	32.3%		36	27.5%		0.73
	2	32	33.3%		47	35.9%		
	3+	33	34.4%		48	36.6%		
TEC external scale		2.37	0.66	1–3	2.27	0.77	0–3	0.021
TEC mental scale		0.76	0.67	0–2	0.88	0.79	0–3	0.007
TEC reflective scale		1.03	0.86	0–3	1.00	0.75	0–3	0.45
TEC total score		4.16	1.56	1–8	4.15	1.46	0–8	0.86

SES, socioeconomic status; BDI, Beck depression inventory; PBI, parental burnout inventory; TEC, test of emotional comprehension; *p*, *p*-value from chi-square and *t*-tests examining the differences between the subsamples of boys and girls.

Parental depression and PB scores were moderately correlated (Pearson correlation coefficient = 0.49, *p* < 0.001).

Table 3 shows that, when adjusted for child age and sex, TEC scales of understanding of external causes of emotions and mental emotions scores were significantly associated with PB. Additionally, we discovered an association of general understanding of emotions with PB in Model 2. While the trends were largely the same, there were no statistically significant associations between parental depression and child emotional development.

**Table 3:** Association of parental depression and burnout with child emotional development.

Predictor	Parental depression						Parental burnout					
	Model 1			Model 2			Model 1			Model 2		
Outcome	*B*	SE	95% CI	*B*	SE	95% CI	*B*	SE	95% CI	*B*	SE	95% CI
TEC external scale	0.11	0.10	−0 0.088; 0.31	0.16	0.11	−0.066; 0.38	**0.17** ^*^	**0.085**	**0.005; 0.34**	**0.20** ^*^	**0.09**	**0.03; 0.37**
TEC mental scale	0.19	0.10	−0.014; 0.39	0.20	0.11	−0.024; 0.42	**0.21** ^*^	**0.09**	**0.043; 0.38**	**0.22** ^*^	**0.09**	**0.05; 0.40**
TEC reflective scale	−0.05	0.11	−0.26; 0.17	0.008	0.07	−0.23; 0.24	−0.046	0.09	−0.22; 0.13	−0.022	0.09	−0.21; 0.16
TEC total score	0.25	0.21	−0.16;0.66	0.36	0.23	−0.090;0.82	0.34	0.17	−0.005; 0.68	**0.40** ^*^	**0.18**	**0.046; 0.75**

*B* refers to unstandardized regression coefficient from multiple regression model; SE refers to standard error; 95% CI refers to 95% confidence interval. Parental depression and burnout score were log-transformed to attain normality for the regression analyses. Model 1 is adjusted for child age at testing and sex. Model 2 is further adjusted for parity, maternal age, level of education, family status, and SES. ^*^p < 0.05. Statistically significant values are highlighted in bold (*p* < 0.05).

Table 4 further shows that when we performed the multiple regression analyses separately for boys and girls, the associations remained significant only for fully adjusted models for total and mental scales and only in girls. Furthermore, we discovered a borderline significant association between parental depression and total emotional development in girls, when controlling for child sex, maternal age, education, family status, socio-economic status (SES), and parity (Table 4).

**Table 4:** Gender dependent association of parental depression and burnout with child emotional development.

Predictor	Boys								Girls							
	Parental depression				Parental burnout				Parental depression				Parental burnout			
	Model 1		Model 2		Model 1		Model 2		Model 1		Model 2		Model 1		Model 2	
Outcome	*B*	95% CI	*B*	95% CI	*B*	95% CI	*B*	95% CI	*B*	95% CI	*B*	95% CI	*B*	95% CI	*B*	95% CI
TEC external scale	0.10	−0.18; 0.38	0.17	−0.16; 0.47	0.18	−0.06; 0.43	0.17	−0.08; 0.42	0.12	−0.16; 0.40	0.15	−0.18; 0.47	0.17	−0.06; 0.40	0.20	−0.05; 0.44
TEC mental scale	0.20	−0.08; 0.49	0.20	−0.13; 0.52	0.21	−0.05; 0.47	0.24	−0.02; 0.49	0.18	−0.10; 0.46	0.24	−0.07; 0.55	0.21	−0.001; 0.44	**0.24** ^*^	**0.003; 0.47**
TEC reflective scale	−0.26	−0.62; 0.10	−0.16	−0.56; 0.23	−0.15	−0.46; 0.16	−0.16	−0.48; 0.16	0.12	−0.14; 0.38	0.20	−0.09; 0.50	0.02	−0.19; 0.24	0.10	−0.12; 0.33
TEC total score	0.05	−0.63;0.72	0.19	−0.58; 0.96	0.24	−0.35; 0.84	0.25	−0.37; 0.87	0.41	−0.10; 0.93	**0.59** ^*^	**0.001; 1.18**	0.41	−0.01; 0.83	**0.54** ^*^	**0.09; 0.98**

*B* refers to unstandardized regression coefficient from multiple regression model; 95% CI refers to 95% confidence interval. Parental depression and burnout score were log-transformed to attain normality for the regression analyses. Model 1 is adjusted for child age at testing. Model 2 is further adjusted for parity, maternal age, level of education, family status, and SES. ^*^p < 0.05. Statistically significant values are highlighted in bold (*p* < 0.05).

However, the interaction analyses revealed that the differences between the subsamples divided by sex were not statistically significant due to low statistical power (*p*-value for all >0.31; observed power <0.17 for all).

## 4. Discussion

The aim of our research was to examine the association between PB, depression and preschoolers’ emotional development.

The obtained data indicate significant correlation between PB and child emotional development. In the present study the association with PB was stronger, than with maternal depression. To date there is a lack of evidence of the PB effects on child emotional development, and our results contribute to this topic. Higher PB scores are associated with higher child outcomes on understanding of the following components of emotion comprehension: external and mental. It is easier for the child to recognize emotions based on facial expressions, to understand external causes of emotions and how memories, desires and beliefs influence emotions. These results contradict our hypothesis of direct adverse effects of PB on child emotional development. PB is associated with parental neglect and violence more often than depression (Mikolajczak et al., 2020; Griffith, 2022). Neglect presumes emotional deprivation, which may be damaging for the child’s emotional development and contribute to internalising problems (Christ et al., 2017; Hunt et al., 2017).

Mothers who are suffering from emotional exhaustion (the dominant PB symptom) report having difficulties in providing clear and consistent emotional responses and have depleted facial expressions of emotions (Roskam et al., 2017). The child has to make an effort to interpret the parent’s state as her safety depends on it and, thus, might develop extra sensitivity. PB is associated with lower parent emotional intelligence and regulation skills (Lin et al., 2022a,b), therefore, the child might develop its own regulation skills to compensate for a parent’s lack of self-regulation. There is data showing that negative effects of PB on child’s mental health can be minimised when parent’s emotional regulation skills are improved (Yang et al., 2021; Lin et al., 2022a,b). In the present study we did not assess parent’s emotional regulation skills, which could play a mediating role and decrease adverse PB effects on children.

According to the cultural-historical framework, parenting is a social construct and parenting behaviour depends on the demands and norms of the society during the historical period (Zakharova, 2012). The social image of the parenting role serves as an orientation system for the style of everyday childcare and parent self-efficiency assessment (Zakharova, 2012; Burmenskaya, 2022). The results obtained in the study may be attributed to the intensive parenting trend and high social demands towards parents and their positive association with PB risks (Roskam et al., 2021). Parents with PB might have invested much effort in the child’s development, which could have led to their exhaustion. Their children, in turn, might have higher TEC scores because of the parent’s past investments. There is also evidence on the role of attachment style in the emotion comprehension development (Cooke et al., 2016; Psychogiou et al., 2018). The parent with PB could manage to develop the secure attachment style, which is associated with better emotion comprehension skills. Moreover, there is data on the significant role of father-child attachment style in the emotion comprehension development (Psychogiou et al., 2018). In our study only mothers participated, and data on fathers’ PB and mental health could enrich the results.

The insignificant association between depression and emotional development contradicts the research accumulated to date (Hentges et al., 2020). This finding might be explained by the characteristics of the sample—there is low awareness of depressive symptoms in Russian society and depression is stigmatised (Dubitskaya, 2009; Beshanova and Kashirskikh, 2020). The study was conducted in the kindergartens, and it might be important for the participants to show the researchers and kindergarten administration that they are competent parents, despite the assurance in the confidentiality of their responses. Therefore, the data may be limited by the social desirability bias. PB is far less stigmatised, so the questionnaire might not elicit socially desirable answers. However, it is important to note that in the present study PB and depression moderately correlate.

One more possible explanation is based on the findings of Nonnenmacher et al. (2021), which indicated no significant associations between theory of mind development and mother’s depression in preschoolers. The authors discovered a mediating role of self-comforting behaviour developed by children, which was a protective factor against an insensitive parenting environment. Children of depressed mothers can develop their own emotion regulation strategies that can decrease the adverse effects of maternal depression.

Our results show moderate gender differences in emotional development: in our study boys scored statistically significantly higher on TEC External scale and lower on TEC Mental scale. Data on gender differences in emotion comprehension is contradictory. There is evidence on girls’ better performance (Bosacki and Moore, 2004; Garner and Waajid, 2008), some studies reveal boys’ better performance in emotion comprehension tasks (Laible and Thompson, 2000), while others indicate no gender differences in emotion understanding (Pons et al., 2004; Denham et al., 2012). Although Aznar and Tenenbaum (2013) found no gender differences in overall TEC scores, boys scored higher than girls in understanding the situational causes of emotion, whereas girls scored higher on understanding reflective emotions. Fidalgo et al. (2018) revealed no gender differences in 8 of 9 TEC components, with the exception of the False belief task, where girls performed better. Thus, our findings support the hypothesis that there are gender disparities in different types of emotion comprehension, but no such trend in total emotional development.

Finally, our study indicated the gender difference in PB and depression effects on emotional development as well: they positively correlate with girls’ emotional development, specifically total TEC scores. PB is also positively related with understanding of mental causes of emotions in girls. The possible explanation of this finding could be related to the characteristics of women’s gender socialisation, as girls are expected to be more sensitive, show empathy and high self-regulation skills (Chaplin and Aldao, 2013). Thereby girls might be more likely to develop understanding of a parent’s emotions as a strategy, compensating for parent’s dysregulation. The 13 years longitudinal study by Murray et al. (2006) also showed that daughters of depressed mothers tend to develop higher sensitivity. Similarly, they demonstrated elevated social maturity. Extra sensitivity was associated with adolescent depression, therefore, the influence of maternal depression can be considered as adverse.

## 5. Conclusion

Our findings support the evidence that PB is distinctive from depression and can have its unique consequences for child emotional development. PB positively correlates with child emotional comprehension skills, specifically understanding of external causes and mental causes of emotions. This effect is gender dependent and is significantly higher for girls. Maternal depression and parental burnout might provoke development of extra sensitivity and self-regulation strategies in girls.

## 6. Limitations

The data obtained in the study might be contaminated by a social desirability bias, because it was collected *via* kindergartens. Despite the confidentiality guarantees, parents could be anxious about revealing their depressive symptoms, as they are socially stigmatised.

Data on PB in fathers could enrich the results and, therefore, further studies that include paternal mental health measures are warranted.

## Data availability statement

The raw data supporting the conclusions of this article will be made available by the authors, without undue reservation.

## Ethics statement

The studies involving human participants were reviewed and approved by Russian Psychological Society. The patients/participants provided their written informed consent to participate in this study.

## Author contributions

AS and VY contributed to conception and design of the study. VY organized the database and wrote sections of the manuscript. AS performed the statistical analysis and edited the manuscript. All authors contributed to the article and approved the submitted version.

## Funding

The research project was supported by the grant of the Russian Science Foundation number 22-18-00356.

## Conflict of interest

The authors declare that the research was conducted in the absence of any commercial or financial relationships that could be construed as a potential conflict of interest.

## Publisher’s note

All claims expressed in this article are solely those of the authors and do not necessarily represent those of their affiliated organizations, or those of the publisher, the editors and the reviewers. Any product that may be evaluated in this article, or claim that may be made by its manufacturer, is not guaranteed or endorsed by the publisher.
